# Male midwifery practice and acceptability: attitudes, beliefs, and associated factors among expectant mothers in Savelugu Municipal Hospital, Northern Region of Ghana

**DOI:** 10.11604/pamj.2024.47.199.42482

**Published:** 2024-04-19

**Authors:** Rashidatu Monne, Mohammed Iddrisu, Joseph Maaminu Kyilleh, Mudasir Mohammed Ibrahim, Abdul Rafiq Nashiru, Abubakari Wuni, Faustina Yin Yariga, Dina Teye-Djangmah, Abdul-Malik Abdulai

**Affiliations:** 1 Nurses´ and Midwives´ Training College, Tamale, Ghana; 2 University for Development Studies, School of Public Health, Social and Behavioral Change, Tamale, Ghana; 3 College of Community Health Nursing Nkanchina, Kpandai, Ghana; 4 Saboba District Health Directorate, Saboba, Ghana; 5 Department of Medicine for the elderly (C6), Cambridge University Hospitals NHS Foundation Trust, Addenbrookes Hospital, Cambridge, United Kingdom

**Keywords:** Male midwifery, beliefs, midwives, expectant mothers, Ghana

## Abstract

**Introduction:**

male midwifery is a relatively new phenomenon in Ghana and most expectant mothers still do not recognize their contribution to reproductive healthcare. This study aims to assess the acceptability of male midwifery practice among expectant mothers in Savelugu Municipal Hospital.

**Methods:**

this was a descriptive cross-sectional study. A total of 391 mothers were recruited using a simple random sampling technique. Data was collected using a structured questionnaire and analyzed using SAS JMP Pro V16.0.

**Results:**

approximately 75.7% of mothers would go to a hospital where male midwives examine and attend to them, and 71.9% accepted to be delivered by a younger male midwife. Also, 70.1% agreed to share their obstetric information with a male midwife, and 43.5% agreed that their religious beliefs allowed them to be delivered by a male midwife. Mothers who had no formal education (aOR=2.23, 95% CI: 1.040-4.788, p=0.039) were more likely to go to a hospital where male midwives examine and attend to them than the others, and mothers who were employed (aOR=3.91, 95% CI: 1.770-8.631, p=0.001) were more likely to accept to be delivered by a male midwife who is younger than them than the others.

**Conclusion:**

a significant portion of expectant mothers are open to receiving care and examinations from male midwives, even opting to go to hospitals where male midwives are available for maternal care. This suggests that male midwives can contribute to the health of expectant mothers significantly and should be encouraged to practice their profession.

## Introduction

Traditionally, the field of maternal health has primarily focused on and been associated with women. Women historically acquired midwifery knowledge through their intuition and personal experiences during pregnancy, as well as by observing others giving birth [1]. This rich history makes midwifery one of the oldest professions globally, dating back to the dawn of human civilization. It was firmly established as a female profession, even in ancient Egypt, as evidenced by the Ebers Papyrus from 1900 to 1500 BCE [2].

The midwifery care model emphasizes that all women, regardless of their backgrounds, deserve safe, effective, and satisfying care throughout their lives. Midwives are now more than ever serving a diverse clientele from various backgrounds, socioeconomic statuses, races, ethnicities, and sexual orientations. Their goal is to provide personalized and attentive care to meet the unique needs of women and their families [3].

The beginning of the midwifery profession can be attributed to a female figure, Florence Nightingale, reinforcing the general perception of it as a women-dominated field [4]. However, there is a growing recognition of the importance of diversifying the midwifery workforce to better cater to clients with diverse demographic characteristics [5]. Modernization has also brought about an increase in the number of men entering the midwifery profession, though their acceptance varies across different countries. For instance, a 2011 study in Great Britain found that only 2% of midwives were male [6,7].

Male midwives face significant challenges, particularly in areas with strong cultural and religious sensitivities [8]. Some countries, like Cameroon, have been encouraging more men to join the midwifery profession due to shortages of healthcare professionals. However, male midwives are not universally accepted, especially in Muslim communities [9]. In Zambia, reports suggest that most women do not prefer male midwives as they perceive them as problematic and oppressive to pregnant patients [10]. Studies conducted in developed countries have indicated a preference among female patients for female midwives due to differences in communication styles between genders, which contributes to higher patient satisfaction with female care providers [11].

Despite these developments, midwifery continues to be predominantly associated with women due to the widespread misconception that it is inherently a female-to-female connection [12]. As stated by Carberinerin, “at all times, as is natural, women have more trust in other women to discover their secrets, problems, and illnesses than they have trust in men” [13]. Midwives do involve male doctors in patient care, sometimes overlooking the shared gender, indicating that various factors contribute to the ongoing lack of gender diversity in midwifery. Potential factors include the absence of male role models for aspiring male midwives and the historical association of nursing with women as a profession [5,14].

In Ghana, efforts have been made to include men in midwifery training to enhance women's access to quality reproductive healthcare. However, this acceptance is more widespread in general nursing, with the field of midwifery being an exception [15]. Despite the increasing number of male midwives, many Ghanaian women do not fully acknowledge their contributions to reproductive healthcare, with some arguing against their presence in the delivery room due to their lack of personal childbirth experience [7].

Male midwifery is a relatively recent development in Ghana, with limited research conducted, particularly in the northern part of the country. There are reports that some expectant mothers do not like to consult male midwives when they are pregnant. Some of them feel uncomfortable consulting male midwives during pregnancy, considering it taboo or inappropriate [16]. The researchers, therefore, designed this study to assess the acceptability of male midwifery practice among expectant mothers in Savelugu Municipal Hospital.

## Methods

**Study design:** a descriptive cross-sectional design (quantitative) was adopted for the study. This design was considered appropriate because it allows for the inclusion of a diverse sample of expectant mothers, enhancing the generalizability of findings to the broader population.

**Study setting:** Savelugu Municipal Hospital is positioned within the Savelugu Municipality, approximately 200 meters away from the Savelugu Customs barrier. The hospital offers 24-hour services including but not limited to in-and out-patient services, antenatal and post-natal services, emergency services, mental health services, laboratory services, and ambulance services. It provides services to residents of the municipal and beyond. The Savelugu municipality shares borders with the Tamale metropolis to the south, Kumbungu to the west, Karaga to the east, and West Mamprusi municipality of the northeast region to the north.

**Study population:** the target population was expectant mothers aged 20-40 years who were seeking antenatal care services at the Savelugu Municipal Hospital. This age bracket was chosen to ensure a blend of women with first-time pregnancies and those who have been pregnant before.

**Inclusion and exclusion criteria:** the inclusion criteria were all expectant mothers aged 20-40 years who expressed free will to participate in the study. The exclusion criteria were expectant mothers outside the 20-40 age bracket and those within the stated age bracket who were unwilling to partake in the study were excluded.

**Sample size and sampling procedure:** the sample size for this study was determined using the Taro Yamane formula for finite populations [17]. With a known population size of 17,082 individuals, a confidence level of 95%, and a margin of error of 5%, the formula below was employed:


$$n=\frac{N}{1+N{\left(e\right)}^{2}}$$

Where; n = sample size, N = population size, and e = margin of error. The resulting calculated sample size was 391. The decision to employ a finite population correction was based on the feasibility of including a significant portion of the population in the study. Expectant mothers were recruited using a simple random sampling approach to ensure a diverse and representative sample. First, a list of expectant mothers eligible for inclusion in the study, who were attending antenatal care services at the Savelugu Municipal Hospital, was compiled. This list served as the sampling frame for the study. Using the JMP Random - sample size generator, a random sample was drawn from the sampling frame.

**Data collection instrument:** a structured questionnaire with closed-ended questions was used for collecting the data. The questionnaire consisted of four sections. The first section comprised of 6 questions to collect data about sociodemographic characteristics. The second section had 8 questions on attitude towards male midwives, the third section had 3 questions on beliefs towards male midwifery practice, and the fourth section had 2 questions on acceptability of male midwifery practice. The instrument was carefully examined by health professionals to ensure face validity. The instrument was also pretested in a different hospital to 15 expectant mothers, and a slight modification was made before the actual data collection process. With the reliability testing, the instrument attained a Cronbach alpha coefficient value of 0.80. This is desirable and satisfactory according to Cortina [18].

**Data collection procedure:** the data for this study were collected through the administration of a structured questionnaire. First of all, the study team formally sought permission from the management of the hospital and the nurse manager of the antenatal care clinic with an introductory letter from the college. Four of our investigators were trained under the supervision of the principal investigator and were responsible for the data collection. They approached the expectant mothers individually, briefly explained the study to them, and invited them to participate voluntarily. Expectant mothers had the free will to participate or not and could opt out during the process at any time. The structured questionnaire was administered face-to-face to expectant mothers who could read and write, allowing them to complete it themselves with guidance from the researchers. They were encouraged to seek clarification on any questions they found unclear. For expectant mothers who could not read and write, the questionnaire was read and translated into the local language, and their responses were recorded as provided. Anonymity was ensured, as names and addresses were not required on the questionnaire for confidentiality purposes. Data collection took place over a eight-week period in the hospital.

**Data management and analysis:** data were organized and cleaned using Microsoft Excel 2021. The data were imported into SAS JMP Pro V16.0 for further analysis. Descriptive statistics were presented using frequencies, percentages, means, and standard deviations. Furthermore, multivariate binary logistic regression was used to measure the association between the acceptability of male midwifery practice and the sociodemographic characteristics of expectant mothers. The level of significance was set at p<0.05 and at a confidence interval of 95%.

**Ethical consideration:** before the actual data collection, ethical clearance was obtained from Savelugu Municipal Hospital (Ref: GHS/NR/RHD/18-0/805). Before giving expectant mothers the questionnaire to fill, written informed consent was obtained and the purpose of the study was explained to them as well. Participation in the study was voluntary. The expectant mothers´ privacy and the data they provided were kept confidential and only used for the research purpose.

## Results

**Sociodemographic characteristics of expectant mothers:** Table 1 showed that most of the expectant mothers fall between the ages of 20-30 years 244 (62.4%). More than half of the expectant mothers were married 271 (69.3%). Also, most of the expectant mothers were Muslims 314 (80.3%). About 147 (37.6%) of them had no formal education. Nearly 302 (77.2%) of the expectant mothers were unemployed.

**Table 1:** sociodemographic characteristics of study participants, recruited from the Antenatal Care Unit of the Savelugu Municipal Hospital (Ghana), from September 2022 to December 2022 (N=391)

Variable	n (%)
**Age**	
20-30 years	244 (62.4)
31-40 years	147 (37.6)
**Marital status**	
Married	271 (69.3)
Single	87 (22.3)
Divorced	33 (8.4)
**Religion**	
Islam	314 (80.3)
Christianity	48 (12.3)
Traditionalist	29 (7.4)
**Education status**	
Junior high school	91 (23.3)
Senior high school	51 (13.0)
Tertiary	102 (26.1)
No formal education	147 (37.6)
**Employment status**	
Employed	89 (22.8)
Unemployed	302 (77.2)

Source: field survey (2022)

**Attitude towards male midwives by expectant mothers:** as presented in Table 2, the majority of expectant mothers, 308 (78.8%) agreed that male midwives are equally skilled professionals as female midwives, 196 (50.1%) agreed that they have trust in female midwives than male midwives, 274 (70.1%) agreed that they would want to share their obstetric information with a male midwife caring for them, 206 (52.7%) agreed that it is okay for women to work as midwives than males, 270 (69.0%) agreed that male midwives are more caring than females, so they will allow them to attend to them. Again, 314 (80.4%) agreed that male midwives are skilled professionals like their female counterparts, so they will allow them to attend to them, 291 (74.5%) agreed that they have trust in male midwives, so they will also accept them to examine and/or assist them to deliver.

**Table 2:** attitude towards male midwives among study participants, recruited from the Antenatal Care Unit of the Savelugu Municipal Hospital (Ghana), from September 2022 to December 2022 (N=391)

Variable	Strongly disagree n (%)	Disagree n (%)	Neutral n (%)	Agree n (%)	Strongly agree n (%)	Mean (SD)
Male midwives are equally skilled professionals as female midwives	2(0.5)	23(5.9)	58(14.8)	120(30.7)	188(48.1)	4.20 ± 0.93
I have trust in female midwives than in male midwives	6(1.5)	65(16.6)	124(31.7)	103(26.3)	93(23.8)	3.54 ± 1.07
I would want to share my obstetric information with a male midwife caring for me	15(3.8)	61(15.6)	41(10.5)	115(29.4)	159(40.7)	3.87 ± 1.21
It is okay for women to work as midwives than males	11(2.8)	68(17.4)	106(27.1)	105(26.9)	101(25.8)	3.55 ± 1.13
Midwifery is a career for females only	42(10.7)	79(20.2)	86(22.0)	108(27.6)	76(19.4)	3.25 ± 1.28
Male midwives are more caring than females, so I will allow them to attend to me	7(1.8)	37(9.5)	77(19.7)	81(20.7)	189(48.3)	4.04 ± 1.11
Male midwives are skilled professionals like their female counterparts, so I will allow them to attend to me	4(1.0)	28(7.2)	45(11.5)	114(29.2)	200(51.2)	4.22 ± 0.98
I have trust in male midwives, so I will accept them to examine and/or assist me to deliver	3(1.0)	44(11.3)	53(13.6)	123(31.5)	168(43.0)	4.05 ± 1.04

Strongly disagree = 1, disagree = 2, neutral = 3, agree = 4, strongly agree = 5; source: field survey (2022)

**Beliefs toward male midwifery practice by expectant mothers:** as shown in Table 3, the majority of the expectant mothers, 170 (43.5%) agreed that their religious beliefs allow them to be delivered by a male midwife, 174 (44.5%) agreed that it is a taboo for male midwives to conduct deliveries, and 168 (43.0%) agreed that their cultural beliefs allow for women to be examined and/or delivered by male midwives.

**Table 3:** beliefs toward male midwifery practice among study participants, recruited from the Antenatal Care Unit of the Savelugu Municipal Hospital (Ghana), from September 2022 to December 2022 (N=391)

Variable	Strongly disagree n (%)	Disagree n (%)	Neutral n (%)	Agree n (%)	Strongly agree n (%)	Mean ± SD
My religious beliefs allow me to be delivered by a male midwife	62(15.9)	73(18.7)	86(22.0)	68(17.4)	102(26.1)	3.19 ± 1.42
It is a taboo for male midwives to conduct deliveries	40(10.2)	88(22.5)	89(22.8)	51(13.0)	123(31.5)	3.33 ± 1.39
My cultural beliefs allow for women to be examined and/or delivered by male midwives	22(5.6)	115(29.4)	86(22.0)	68(17.4)	100(25.6)	3.28 ± 1.28

Strongly disagree = 1, disagree = 2, neutral = 3, agree = 4, strongly agree = 5; source: field survey (2022)

**Acceptability of male midwifery practice by expectant mothers:** Table 4 presents that a greater number of expectant mothers, 296 (75.7%) would go to a hospital where male midwives examine and attend to mothers, and 281 (71.9%) accepted to be delivered by a male midwife who is younger than them.

**Table 4:** acceptability of male midwifery practice among study participants, recruited from the Antenatal Care Unit of the Savelugu Municipal Hospital (Ghana), from September 2022 to December 2022 (N=391)

Variable	n (%)
**Would you go to a hospital where male midwives examine and attend to expectant mothers?**	
Yes	296 (75.7)
No	95 (24.3)
**Would you accept to be delivered by a male midwife who is younger than you?**	
Yes	281 (71.9)
No	110 (28.1)

Source: field survey (2022)

**Factors associated with acceptance to go to a hospital where male midwives examine and attend to mothers:** as illustrated in Table 5, the multivariate logistic regression analysis revealed that expectant mothers who were divorced (aOR=3.93, 95% CI: 1.313-11.772, p=0.014), who were married (aOR=3.88, 95% CI: 2.023-7.460, p<0.001), who had no formal education (aOR=2.23, 95% CI: 1.040-4.788, p=0.039), and who were employed (aOR=2.25, 95% CI: 1.074-4.751, p=0.031) were more likely to go to a hospital where male midwives examine and attend to them than the others.

**Table 5:** factors associated with acceptance to go to a hospital where male midwives examine and attend to mothers among study participants, recruited from the Antenatal Care Unit of the Savelugu Municipal Hospital (Ghana), from September 2022 to December 2022 (N=391)

Variable		Adjusted odds ratio	P-value	95% confidence interval (Wald)	
				Lower	Upper
Age	31-40 years	Ref	0.396		
	20-30 years	0.78		0.447	1.374
Marital status	Single	Ref			
	Divorced	3.93	0.014*	1.313	11.772
	Married	3.88	< 0.001*	2.023	7.460
Religion	Traditionalist	Ref			
	Christianity	0.706	0.599	0.192	2.585
	Islam	0.612	0.393	0.198	1.890
What is your level of education?	Tertiary	Ref			
	Junior high school	1.22	0.608	0.570	2.609
	No formal education	2.23	0.039*	1.040	4.788
	Senior high school	2.11	0.114	0.834	5.369
What is your employment status?	Unemployed	Ref	0.031*		
	Employed	2.25		1.074	4.751

* Statistically significant at p<0.05

**Factors associated with acceptance to be delivered by a male midwife who is younger than them:** as displayed in Table 6, the results of multivariate logistic regression analysis revealed that expectant mothers who were divorced (aOR=3.34, 95% CI: 1.249-8.962, p=0.016), who were married (aOR=3.78, 95% CI: 1.980-7.233, p<0.001), who were Christians (aOR=4.62, 95% CI: 1.517-14.120, p=0.007), who were Muslims (aOR=5.80, 95% CI: 2.294-14.666, p=0.001), who had no formal education (aOR=4.01, 95% CI: 1.819-8.853, p=0.001), and who were employed (aOR=3.91, 95% CI: 1.770-8.631, p=0.001) were more likely to accept to be delivered by a male midwife who is younger than them than the others.

**Table 6:** factors associated with acceptance to be delivered by a male midwife who is younger than them among study participants, recruited from the Antenatal Care Unit of the Savelugu Municipal Hospital (Ghana), from September 2022 to December 2022 (N=391)

Variable		Adjusted odds ratio	P-value	95% confidence interval (Wald)	
				Lower	Upper
Age	31-40 years	Ref	0.217		
	20-30 years	1.40		0.818	2.404
Marital status	Single	Ref			
	Divorced	3.34	0.016*	1.249	8.962
	Married	3.78	< 0.001*	1.980	7.233
Religion	Traditionalist	Ref			
	Christianity	4.62	0.007*	1.517	14.120
	Islam	5.80	0.001*	2.294	14.666
What is your level of education?	Tertiary	Ref			
	Junior high school	2.03	0.077	0.924	4.475
	No formal education	4.01	0.001*	1.819	8.853
	Senior high school	0.98	0.979	0.429	2.276
What is your employment status?	Unemployed	Ref	0.001*		
	Employed	3.91		1.770	8.631

* Statistically significant at p<0.05

## Discussion

This research assessed the acceptability of male midwifery practice among expectant mothers in Savelugu Municipal Hospital. In this study, most of expectant mothers (78.8%) expressed agreement that male midwives are equally skilled professionals as female midwives. This observation is in line with the reports from Zambia and Kenya [2,19]. These two previous studies found that expectant mothers acknowledged that male midwives are equally skilled as compared to female midwives. Also, another study from Nigeria reported that the majority of expectant mothers were in support of the view that male midwives are equally skilled alongside female midwives [14].

Within the scope of this study, 71.9% of expectant mothers have trust in male midwives, and are open to accepting them to examine and/or assist them to deliver. These results are consistent with prior research from Zambia and Ghana where many expectant mothers willingly accepted the services of male midwives due to their training and the comparable level of care they provided when compared to their female counterparts [7,9]. This consistency may be due to the frequent interaction that expectant mothers have with male midwives, which is likely to positively influence their attitudes towards male midwifery [20].

The current study revealed that more than half of expectant mothers (52.7%) agreed that it is okay for women to work as midwives than males, and this was in line with a past study from Zambia [19]. Furthermore, two studies from Turkey have shown that expectant mothers supported the notion that only women should pursue midwifery and provide obstetrics services [6,21].

This study revealed that a significant portion of expectant mothers (43.5%) expressed agreement with the idea that their religious beliefs allowed them to deliver and receive care from a male midwife. This finding is particularly interesting when examining the attitudes of expectant mothers towards the practice of male midwifery. This is noteworthy given that, in the study locale, a predominant majority are Muslims and Islamic law strictly prohibits male doctors or midwives from conducting examinations or touching the exposed bodies of women, particularly in intimate areas. The provision of care to women is exclusively entrusted to female midwives, with rare exceptions allowed only in cases where the life of the mother and/or the fetus is in jeopardy. It aligns with the study results from Zambia, which found that about 36.5% of expectant mothers agreed that their religious beliefs permitted them to be attended to by a male midwife [19]. It is worth noting that this agreement might not be solely attributed to religious beliefs, as the majority of expectant mothers in this current study were Muslims (80.3%), whereas that of the study from Zambia were Christians. The current result, however, is not in agreement with the study from the United Kingdom which showed that expectant mothers generally preferred female midwives over male midwives due to religious reasons [22]. Furthermore, according to a study from Egypt, even male student midwives faced challenges of rejection and lack of cooperation from expectant mothers [23].

In the current study, nearly 44.5% of expectant mothers expressed the view that it is inappropriate for male midwives to assist with childbirth, which is in line with the findings from Zambia [19]. Additionally, the study revealed that 43.0% of expectant mothers believed that their cultural perspectives allowed for women to be attended to and examined during labor by male midwives, and this was not in line with the research from Nigeria [14]. These findings indicate that cultural beliefs and religious considerations can impact antenatal care services and childbirth practices in Ghana, alongside the educational and professional backgrounds of mothers and male midwives, respectively.

The research findings indicated a significant majority of expectant mothers (76.2%) were willing to be delivered or examined by a male midwife. Moreover, about 75.7% of them accepted to go to a hospital where male midwives examine and attend to mothers, and 71.9% accepted to be delivered by a male midwife who is younger than them. This corroborates with the study from Zambia where 83% of expectant mothers approved of receiving treatment from male midwives, citing their equal training and access to resources as female midwives [10]. In contrast, another study from Zambia reported a potential delay in the acceptance of males as midwives, with expectant mothers expressing reluctance to deliver at a health center where a male midwife conducted deliveries and disagreeing with the idea of having their babies delivered by a younger male midwife (59.0%, 63.0% respectively) [19]. A separate survey from Turkey also revealed that a majority of expectant women believed midwifery was a field more suitable for women and expressed a preference not to have male midwives involved in their care during pregnancy and labor [22].

**Limitations:** this research utilized only one district hospital in Ghana´s Northern Region which may not be an accurate representation of expectant mothers in the region. Also, due to a dearth of research in the field, it was difficult to compare results with other studies both local and international. Lastly, from the expectant mothers´ perspective, there could be over-reporting of the situation since the study relies on self-reported measurements.

## Conclusion

The findings of the study reveal that a considerable number of expectant mothers do acknowledge the existence of societal taboos surrounding male midwives conducting deliveries, as well as recognizing that their cultural norms traditionally permit female midwives to perform such roles. However, a significant portion of expectant mothers are open to receiving care and examinations from male midwives, even opting to go to hospitals where male midwives are available for maternal care. This suggests that male midwives can contribute to the health of expectant mothers significantly and should be encouraged to practice their profession.

### What is known about this topic

- *The inception of the midwifery profession can be traced to Florence Nightingale, a female figure, thereby reinforcing the prevalent perception of it as a female-dominated field;*
- *Male midwifery is a relatively recent phenomenon in Africa, and a majority of expectant mothers still do not acknowledge the significant role that male midwives play in reproductive health care;*
- *Male midwives encounter substantial challenges, especially in regions characterized by strong cultural and religious sensitivities*.

### What this study adds

- *Expectant mothers perceive male midwives as more compassionate than their female counterparts, leading them to willingly seek their care;*
- *Expectant mothers express a willingness either to choose hospitals where male midwives conduct examinations and provide care or to opt for delivery under the guidance of a younger male midwife;*
- *Expectant mothers who are divorced, married, lack formal education, or are employed are more inclined to choose hospitals where male midwives conduct examinations and provide care, highlighting the significance of these factors in shaping maternal healthcare decisions*.
